# Does clinical teacher training always improve teaching effectiveness as opposed to no teacher training? A randomized controlled study

**DOI:** 10.1186/1472-6920-14-6

**Published:** 2014-01-08

**Authors:** Jan Breckwoldt, Jörg Svensson, Christian Lingemann, Hans Gruber

**Affiliations:** 1 Medical Faculty of the University of Zurich, Pestalozzistr. 3-5, Zurich CH-8091, Switzerland; 2 Department of Anaesthesiology, Charité, Medical University of Berlin Campus, Benjamin Franklin, Berlin, Germany; 3 Department of Educational Science, University of Regensburg, Regensburg, Germany; 4 Dieter Scheffner Centre for Medical Education, Charité – Medical University of Berlin, Berlin, Germany

**Keywords:** Expertise, Faculty development, Standardized clinical examination, Teacher training, Teaching effectiveness

## Abstract

**Background:**

Teacher training may improve teaching effectiveness, but it might also have paradoxical effects. Research on expertise development suggests that the integration of new strategies may result in a temporary deterioration of performance until higher levels of competence are reached. In this study, the impact of a clinical teacher training on teaching effectiveness was assessed in an intensive course in emergency medicine. As primary study outcome students’ practical skills at the end of their course were chosen.

**Methods:**

The authors matched 18 clinical teachers according to clinical experience and teaching experience and then randomly assigned them to a two-day-teacher training, or no training. After 14 days, both groups taught within a 12-hour intensive course in emergency medicine for undergraduate students. The course followed a clearly defined curriculum. After the course students were assessed by structured clinical examination (SCE) and MCQ. The teaching quality was rated by students using a questionnaire.

**Results:**

Data for 96 students with trained teachers, and 97 students with untrained teachers were included. Students taught by untrained teachers performed better in the SCE domains ‘alarm call’ (p < 0.01) and ‘ventilation’ (p = 0.01), while the domains ‘chest compressions’ and ‘use of automated defibrillator’ did not differ. MCQ scores revealed no statistical difference. Overall, teaching quality was rated significantly better by students of untrained teachers (p = 0.05).

**Conclusions:**

At the end of a structured intensive course in emergency medicine, students of trained clinical teachers performed worse in 2 of 4 practical SCE domains compared to students of untrained teachers. In addition, subjective evaluations of teaching quality were worse in the group of trained teachers. Difficulties in integrating new strategies in their teaching styles might be a possible explanation.

## Background

Teacher training may improve quality and professionalism of teaching and is therefore widely employed in faculty development programmes [1-9]. However, training may have the paradoxical effect of reducing actual performance. Research on expertise development suggests that the integration of new strategies could result in deterioration of performance until higher levels of competence are reached. As a result of new learning, current routines, procedures, and organization of knowledge, which had worked quite well before the training, may be called into question. In particular, meta-cognitive components of training may make learners aware of their potential for further development [10-12]. This awareness may reveal one’s own deficits to the trainee, and may therefore cause insecurity [13].

Furthermore, developing and integrating new strategies into a routine requires considerable time, re-training and practice [14], often referred to as deliberate practice [15]. As a consequence, during an intermediate time the training might therefore lead to detrimental effects. Evidence exists that such U-shaped developments occur while new information or new skills are being worked into an existing teaching routine: When new paths are entered, some drawbacks in performance are to be expected. In the field of clinical teacher training however, this potential phenomenon has not yet been clearly addressed. We therefore wanted to explore the impact of clinical teacher training at an early time point after intervention. To measure quality and effectiveness of teaching we chose teachers’ performance in an intensive course in emergency medicine. Using a prospective randomized controlled design, the investigators were blinded towards teacher training and teachers were blinded towards the final purpose of the study. At present, the Objective Structured Teaching Exercise (OSTE) is the most extensively studied instrument to assess teaching quality [16]. However, we preferred to use a different outcome parameter since the ultimate goal of teaching in emergency medicine is to improve students’ skills and the outcomes for patients. With these goals in mind, we chose practical resuscitation skills as the primary outcome parameter. We assessed:

•students’ practical and theoretical performance at the end of the emergency medicine course,

•teaching quality rated via evaluation via participating students, and

•self-perception of teachers.

## Methods

In August 2006 eighteen clinical teachers from emergency medicine and anaesthesiology were matched with respect to their clinical experience and teaching experience, and then randomly assigned to a session of teacher training lasting two days (‘trained group’) or no training (‘untrained group’). In anticipation of organizational shortcomings within the department, we planned one additional pair of teachers as backup. From October 2006 to June 2007, all of the teachers in the sample were routinely scheduled to teach emergency medicine to 3^rd^ year students. [In Germany, medical students start their curriculum after 13 years of school. Their first two years at university predominantly cover basic science].

### Characteristics of teachers

All clinical teachers were employees of a university teaching hospital. None had received any teacher training prior to the study. For each emergency medicine course, a team of three teachers was responsible, at three different levels of clinical experience. These levels were defined by the learning content, which each teacher had to cover in his or her specific sessions: ‘juniors’ had a clinical experience of 0–1 years (in clinical anaesthesiology), ‘intermediates’ 3–6 years (additional experience in intensive care and emergency room service), and ‘seniors’ 7 or more years (additional experience in pre-hospital emergency medicine). In terms of teaching experience, the ‘junior’ group did not have any, ‘intermediates’ had 1–4 years, and ‘seniors’ had 5 or more years.

### Teacher training

Teachers who were assigned to the intervention group attended a two-day clinical teacher training, which was part of the faculty development programme of the Charité –Medical University of Berlin. Training was carried out by one to two experienced educators with a background in educational psychology (generic adult learning) or clinical teaching (clinical application). The learning contents were: ‘role of the teacher’, ‘needs of learners’, ‘providing feedback’, ‘structure of session’, ‘defining learning objectives’, ‘activating learners’, ‘teaching of skills’, ‘teaching with patients’. The courses contained 10–14 participants, the training involved discussion groups, role-play, and reflection exercises (for details see Additional file 1: Table S1). The training was well received, attaining an overall rating by participants of 85-90% of the possible maximum.

Teaching sessions for the assessment of teaching performance after the training were scheduled two to three weeks later.

### Emergency medicine course

The course of 12 full hours within two days followed a pre-defined structure. For a total of 16–18 students, each course involved a team of three teachers (junior, intermediate, senior). After an introductory interactive plenary session, students were divided into groups of 5–6. These groups rotated through 6 teaching sessions, each of which was taught by one member of the teacher team. Teaching formats were based on scenario teaching and discussion groups and focussed on the acquisition of practical skills. Learning objectives (in accordance with the guidelines of the European Resuscitation Council) were: ‘initial assessment of the emergency patient’, ‘management of unconsciousness’, ‘basic life support (BLS)’, ‘use of automated external defibrillator (AED)’, ‘bag-mask-ventilation’, and ‘basic trauma management’. There was a formative assessment of BLS at the end of day 1 and a three-station standardized clinical examination (SCE) at the end of day 2. The SCE was mandatory to pass the course, but failure rates were known to be low from previous student cohorts.

### Characteristics of students

We included only data from students who regularly completed the entire emergency medicine course and who were not enrolled in any external students’ exchange program.

### Measures of evaluation

#### Assessment of student skills and knowledge

After the emergency medicine course student performance was assessed by a three-station SCE (structured clinical examination) and an MCQ test. The SCE was the official assessment at the end of the course, while the MCQ was an additional test.

From the SCE, we extracted four domains of skills, which were relevant within the test setting. This was done because scenario testing does not necessarily exhibit good content validity for *all* checklist items (e.g. ‘check for safety’ cannot easily be assessed under the safe conditions of an assessment situation). To enhance clinical relevance we only chose skills related to patient safety based on international resuscitation guidelines. Skill domains were: ‘alarm call’, ’chest compressions’, ‘use of AED’, ‘bag-mask ventilation’. Each domain was analysed separately. For the total SCE score single domains were summed up.

The MCQ included 14 single-choice questions, covering relevant content of the course, partly factual, partly procedural knowledge. We limited the number of questions for reasons of time constraints and to ensure students’ acceptance. The MCQ was validated by comparison with final year students within their anaesthesia clerkship (with 97% of answers being correct).

#### Evaluation of teachers by students

The 30-item questionnaire was based on a review of the literature on teaching quality in medical education [5,17-22]. In accordance with two reviews in this field [17,20], we also linked our questions to two main fields of teaching performance: ‘interpersonal communication’ and ‘structural aspects of teaching’. Ten questions dealt with student-teacher interaction, and 13 questions with teaching structure. Also, general acceptance of individual clinical teachers was rated (questionnaire see Additional file 2: Figure S1). Answers were given on a 7-point Likert-like scale (from +3 ‘strongly agree’ to −3 ‘strongly disagree’).

#### Teacher self-perception

Teachers reported their perceptions of competence, self-confidence, and their overall satisfaction with their teaching session on a 7-point Likert-like scale.

### Informed consent

All individual data of students and teachers were transformed into a pseudonymous format for all further steps of data processing to ensure that individuals could not be identified later. Teachers and students were formally asked to participate with the option to prohibit the use of their data at any time. All gave verbal consent. They were told that the study aimed to explore the effect of the composition of the group of teachers. In order to minimize instrumentation bias they were not informed that the focus of the study was the teacher training. Approval was given by the educational board of Charité – Medical University of Berlin.

### Statistical analysis

The sample size was calculated for a 15% difference of the total score of all four SCE domains. At a power level of 0.9 with an assumed alpha of 0.05, the sample size was set at 75 students per group.

Results of SCE and MCQ were transformed into percentages of the achievable maximum, and then medians, 25^th^ and 75^th^ percentiles were calculated. Groups were compared using a Mann–Whitney-U-test. Significance was assumed at p-values below 0.05. The analyses were performed using SPSS 14.0.

## Results

### Characteristics of students

From a total of 210 students, 17 were excluded from analysis (13 students from external exchange programmes, 4 with incomplete attendance). From the remaining 193 students, 96 were taught by trained teachers, and 97 by untrained teachers. No statistical differences were found between the groups regarding age (median: 24 vs. 23 years), female gender (56.3% vs. 59.6%), German as native language (85.4% vs. 86.6%), or previous experience in emergency medical services (13.7% vs. 11.3%), for details see Additional file 3: Table S2.

### Teacher characteristics

All student courses were run exclusively with either trained teacher teams or untrained teams. Furthermore, all courses were taught by one junior, one intermediate, and one senior teacher. Due to clinical shifts, department rotations, and one teacher leaving the institution, the matched pair design could not be kept up entirely: three trained teachers did not teach within the context of the study, and one untrained teacher had to be recruited as an additional control.

Trained teachers taught a median of 3 courses (range 1–5), untrained teachers a median of 2 (1–4) courses. Time between training and first teaching encounter was 14 days in median (range 6–41). Self-reported motivation to teach was “high” to “very high” and did not differ between the groups. Resulting teacher characteristics are shown in Table 1, the final study composition is shown in Additional file 4: Figure S2.

**Table 1:** Characteristics of individual clinical teachers

**Teachers**	**Gender**	**Time between training and first teaching**	**Clinical experience (in years)**	**Teaching experience (in years)**	Motivation to teach*	**Number of courses taught**
Junior pair 1	f	15 days	1.5	0	+3	1
	f	-	1	0	+2.5	3
Junior pair 2	m	7 days	< 1	0	+3	5
	m	-	< 1	0	+3	2
Junior pair 3	f	Training but no teaching				
	f	-	< 1	0	+3	2
Intermediate pair 1	f	6 days	5	3	+2	2
	f	-	5	3	+3	4
Intermediate pair 2	m	27 days	5	3	+3	4
	m	-	5	3	+2.5	2
Intermediate pair 3	f	Training but no teaching				
	f	-	4	2	n.a.**	1
Senior pair 1	m	13 days	8	6	+3	3
	m	-	8	6	+3	2
Senior pair 2	f	41 days	9	6	+2	3
	f	-	8	6	+3	2
Senior pair 3	m	Training but no teaching				
	m	-	7	5	+3	2
Addit. control	m	-	10	7	+2	1

*Measured an a 7-point Likert scale (‘how much do you appreciate teaching in general?’ from −3: ‘not at all’ to +3: ‘very strongly’).

**Not answered.

### Test results of students

#### Practical skills: Standardized clinical examination (SCE)

**Figure 1:**
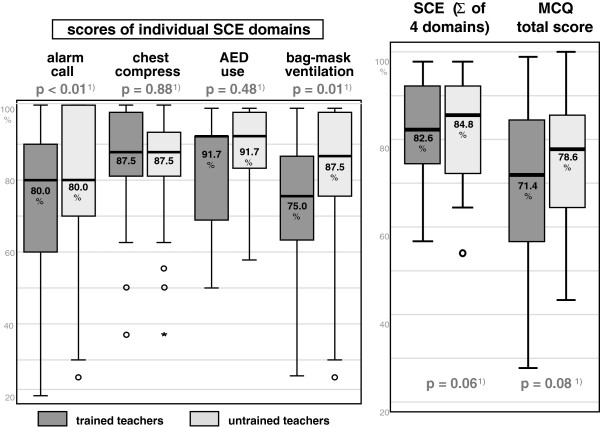
Students’ practical (SCE) and theoretical (MCQ) test results.

Four clinically relevant domains were extracted from SCE for analysis. The domains ‘chest compressions’ and ‘use of AED’ did not show significant differences (see Figure 1). Notably in these domains both groups of students scored very high.

For the domains ‘alarm call’ and ‘bag-mask ventilation’ students with untrained teachers scored significantly better than the trained group (p < 0.01 and p = 0.01).

The overall SCE score was better for the untrained teacher group, but statistical significance was not reached (p = 0.06). Cronbach’s Alpha for the sum of all four SCE domains was 0.77.

#### Theoretical knowledge: MCQ

In the MCQ, students of trained teachers gave correct answers in a median of 71.4% (57.1-83.9%, 25^th^-75^th^ percentile), while students from untrained teachers reached a median of 78.6% (64.3-85.7%). No statistical difference could be shown between the groups (p = 0.075), see Figure 1. For six single questions, results were statistically different, for five of these in favor of the untrained teacher group. Questions representing procedural knowledge showed no significant difference (Additional file 5: Table S3). Internal test consistency was calculated with a Cronbach’s Alpha of 0.53.

### Evaluation of clinical teachers by students

**Figure 2:**
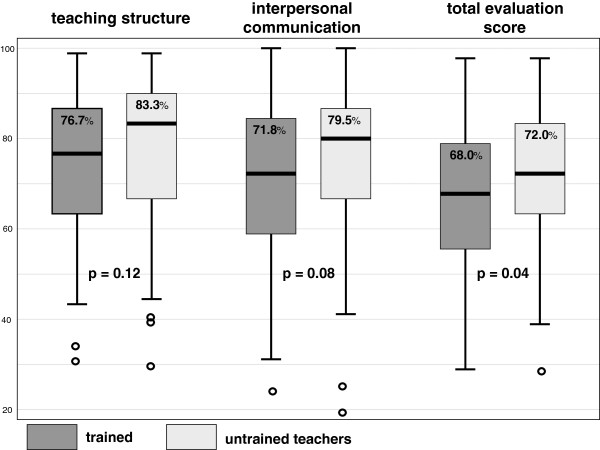
Evaluation of teaching quality by students.

1) For the sum of all items for teaching quality, students of the trained teachers gave a significantly lower rating than the students of untrained teachers (68.0% (56.0-79.0%) vs. 72.0% (63.0-83.0%), p = 0.04, see Figure 2).

2) For the sub-group of structural teaching aspects and for interpersonal communicative performance no statistical difference could be shown, but in both sub-domains a tendency towards lower ratings for the trained teachers was seen (teaching structure: 76.7% (63.3-86.7%, 25^th^-75^th^ percentile) vs. 83.3% (66.7-90.0%, p = 0.12; interpersonal communication: 71.8% (59.0-84.6%) vs. 79.5% (66.7-87.2%) p = 0.08), see Figure 2).

3) Results of the global rating of individual teachers are shown in Table 2. The group of trained ‘senior’ teachers again was rated significantly lower than the untrained seniors (2.57 vs. 2.74, p < 0.01). For ‘juniors’ and ‘intermediates’ no significant differences were shown.

**Table 2:** Evaluation of individual teachers by students (global performance rated on a 7-point Likert-like scale from −3 ‘very bad’ to + 3 ‘very good’)

**Global performance of**		**With training (n = 96)**	**Without training (n = 97)**	**p-value**
**Junior teacher**	Mean	**2.17**	**2.28**	n.s.
	Median	**2** (2–3)	**3** (1–3)	n.s.
**Intermediate teacher**	Mean	**2.64**	**2.71**	n.s.
	Median	**3** (2–3)	**3** (2–3)	n.s.
**Senior teacher**	Mean	**2.57**	**2.74**^1)^	p = 0.008
	Median	**3** (2–3)	**3** (3–3)^1)^	n.s.

^1)^n = 95.

### Self-perception of teachers

Results are shown in Table 3 as qualitative data because every distinct category only included 6–7 ratings. Trained teachers appeared to feel more competent and more self-confident. This effect seems to be strongest for the ‘intermediate’ teachers. Overall satisfaction with the teaching session did not differ between the groups.

**Table 3:** Self perception of teachers

**Question**	**Teacher’s clinical competence**	**Trained**	**Untrained**
**How competent did you feel in your session?**	Junior	**2.0** (6)^1)^	**1.7** (7)
	Intermediate	**2.5** (6)	**1.9** (7)
	Senior	**2.2** (6)	**2.0** (7)
	All competence levels	**2.2** (18)	**1.9** (21)
**How confident/sure of yourself did you feel during your session?**	Junior	**2.1** (6)	**1.8** (7)
	Intermediate	**2.5** (6)	**2.0** (7)
	Senior	**2.2** (6)	**2.3** (7)
	All competence levels	**2.3** (18)	**2.0** (21)
**How satisfied did you feel with your session?**	Junior	**1.5** (6)	**1.8** (7)
	Intermediate	**2.0** (6)	**1.7** (7)
	Senior	**1.9** (6)	**1.8** (7)
	All competence levels	**1.8** (18)	**1.8** (21)

^1)^Mean ratings on a 7-point Likert-like scale from −3: ‘not at all’ to + 3 ‘very much/strong’; in brackets: number of evlauations.

## Discussion

In this study none of the measured parameters of teaching performance was found to be superior in the group of trained teachers. This finding specifically refers to the primary endpoint (practical skills of students), where two domains even revealed a significantly worse performance in the trained teacher group. Looking at the two other domains more closely (chest compressions and AED use), it has to be considered that students reached very high scores. This might indicate a ceiling effect, which can mask potential differences. On the other hand teachers might have strongly focussed on these core competencies in resuscitation, since they hold the highest evidence level in international resuscitation guidelines.

The secondary endpoint ‘theoretical knowledge’ (MCQ) also showed no advantage of the trained group with a tendency towards inferior test results for the group of trained teachers. Evaluation of teaching quality by students revealed significantly lower ratings for trained teachers, with analogous trends for the sub-domains ‘teaching structure’ and ‘interaction’.

As stated at the beginning of this article, the most likely explanation for these negative results are difficulties in newly trained teachers putting acquired theoretical concepts into practice. After a two-day training with plenty of information and a generally strong shift in attitudes (from teacher- to learner-centeredness), teaching performance was assessed when the learners had not yet been given time to practice the newly-learned techniques. Thus a ‘confusion effect’ may have occurred, making it impossible to adequately integrate the contents of the training, resulting in poorer teaching performance. This hypothesis may be supported by the self-perception of trained teachers, who did not feel more satisfied with their sessions although they reported higher levels of competence and self-confidence. A similar phenomenon was described in the field of business school education, where business school students performed inferior to untrained controls, when they had to transfer their substantial knowledge to practical applications [13,23].

One might speculate that teaching quality would have improved if it had been assessed at a later time. Unfortunately re-evaluation of teachers was not possible because some of the teachers had since left the institution.

Our findings indicate that duration and timing – the “dosage” – of teacher training need to be considered carefully. Other training programmes of longer duration have been proven to be efficient [5,24], and even some shorter programmes have improved teaching performance [6,9,25]. However, these programmes were predominantly designed for resident and clerkship teaching, and teaching quality was assessed at a later time point long after training, offering considerable practical opportunity to integrate new competencies.

Another aspect might be the distribution of training over time. While many courses are run as one or two-day seminars or workshops, Morrison et al. spread their training of only 13 hours over 6 months and substantially improved the score in an objective standardized teaching examination [3]. In this light, shorter training units might be more “digestible”, hence in our two-day training we might have administered an “overdose”. However, whether teaching performance might have improved over time remains an open question.

### Potential limitations

#### Measurement validity

For the primary endpoint, the students’ practical test results, good validity can be assumed, since the applied SCE domains had good content validity and good internal consistency. Furthermore, the calculated sample size was reached.

Regarding MCQ results, internal consistency of the scale was low, most likely due to the small number of items. This restriction followed practical reasoning in order to keep the students’ workload tolerable. We also acknowledge that while observing a group effect, the low Cronbach’s Alpha value allows only preliminary interpretations.

As a further limitation, the questionnaire for teacher evaluations was not validated. Nevertheless it was supported by sound literature and was specifically tailored to the study setting. Sub-analysis was also supported by the literature [17,20]. The gender distribution of teachers was imbalanced, with a larger number of female teachers. Research, however, does not suggest a substantial effect of gender on teaching quality [26,27]. Factors such as teaching experience probably play a greater role.

#### Appropriateness of the teacher training for the teaching format

Although there is little standardization of teacher training, we assume for our training that learning contents and qualifications of educators met common standards. Learning contents covered at least 75% of the teaching situations, which explicitly occurred in the studied emergency medicine course (Additional file 1: Table S1). Perhaps the training may have shifted the teachers’ focus from pure skill training to a more holistic teaching approach fostering deep learning. In this case, the practical skills of students might not represent a relevant outcome for contents delivered by the training. However, evaluation of teaching quality by students (including student-teacher interaction) did not indicate a more learner-centred attitude of trained teachers. The impact of the intervention is additionally supported by the trained teachers reporting a higher degree of self-confidence.

The pre-structured format of the emergency medicine course might itself have had an influence on the teaching quality providing a curricular setting for effective learning. Notably, all clinical teachers achieved high ratings. Further reasons for favourable ratings could have been high initial teaching competencies [28] and the fact that students strongly value the practical teaching in emergency medicine [29]. However, these points would not explain the inferior teaching performance of trained teachers. Nonetheless, the curricular structure might have hampered the individual creativity of teachers by defining learning objectives, materials and methods in advance.

#### Generalizability

Primarily the study results are restricted to a single institution since the teacher training may be not comparable to others. Nonetheless, we think that the main finding of this study – the lack of improvement in the trained teacher group – is consistent over the different study outcomes. This finding appears important, since the mechanisms of acquisition of competencies from teacher training sessions are not well understood. It is possible that specific circumstances of the study design brought to sight an early stadium of transition during learning of complex skills. The results of this study should advise us to think more thoroughly about timing, duration, and amount of content of teacher trainings. At least, it could be of value to know about the possibility that teaching performance does not necessarily improve immediately after training. Further investigations should explore this field in more depth and focus on the longitudinal analysis of teacher development.

## Conclusions

In this prospective study, outcomes after clinical teacher training teaching were not found to be superior compared to untrained controls. For a number of study end points, results even turned out to be significantly worse. A potential reason could be that trained teachers experienced difficulties in integrating new teaching competencies into their teaching practice at this time point. Further research is warranted to clarify the underlying mechanisms.

## Abbreviations

SCE: Standardized clinical examination; MCQ: Multiple choice questionnaire; AED: Automated external defibrillator; BLS: Basic life support.

## Competing interests

The authors declare that they have no competing interests.

## Authors’ contributions

JB designed the study and the teaching concept, and prepared the manuscript. JS collected the data and conducted statistical calculations. CL collected data, contributed to the study design and manuscript. HG made important intellectual contributions to the study design and to the manuscript. Each author takes public responsibility for the entire work. All authors read and approved the final manuscript.

## Authors’ information

JB was clinical educator in anaesthesiology in association with Dieter Scheffner Center for Medical Education at Charité – University Medicine Berlin, Germany and is now coordinator at the Vice Deanery of Education of the Medical Faculty at the University of Zurich, Switzerland. JS is a former student at the dept. of anaesthesiology, now a physician in radiology at Vivantes Hospitals, Berlin, Germany. CL is a former student of the dept. of anaesthesiology, now a physician at the Dept. of Cardiology, Hospital of the German Red Cross, Berlin-Westend. HG is full professor of educational science, Head of the Department of Educational Science, University of Regensburg, Germany.

## Pre-publication history

The pre-publication history for this paper can be accessed here:

http://www.biomedcentral.com/1472-6920/14/6/prepub

## Supplementary Material

Additional file 1: Table S1 Structure of teacher training (overview). Click here for file

Additional file 2: Figure S1 Questionnaire for evaluation by students. Click here for file

Additional file 3: Table S2 Characteristics of students. Click here for file

Additional file 4: Figure S2 Resulting final study configuration. Click here for file

Additional file 5: Table S3 MCQ by single questions. Click here for file

## References

[B1] Frohna AZ Hamstra SJ Mullan PB Gruppen LD Teaching medical education principles and methods to faculty using an active learning approach: the university of Michigan medical educators scholars program Acad Med 2006 81 975 978 10.1097/01.ACM.0000242573.71314.74 17065859

[B2] Hewson MG A theory based faculty development program for clinician-educators Acad Med 2000 75 498 501 10.1097/00001888-200005000-00024 10824777

[B3] Morrison EH Rucker L Boker JR Gabbert CC Hubbell FA Hitchcock MA Prislin MD The effect of a 13-hour curriculum to improve residents’ teaching skills Ann Intern Med 2004 141 257 263 10.7326/0003-4819-141-4-200408170-00005 15313741

[B4] O’Sullivan PS Irby DM Reframing research on faculty development Acad Med 2011 86 421 428 10.1097/ACM.0b013e31820dc058 21346505

[B5] Skeff KM Stratos G Berman J Bergen MR Improving clinical teaching – Evaluation of a national dissemination program Arch Intern Med 1992 152 1156 1161 10.1001/archinte.1992.00400180028004 1599342

[B6] Steinert Y Mann K Centeno A Dolmans D Spencer J Gelula M Prodeaus D A systematic review of faculty development initiatives designed to improve teaching effectiveness in medical education: BEME guide No. 8 Med Teach 2006 28 497 526 10.1080/01421590600902976 17074699

[B7] Steinert Y McLeod PJ From novice to informed educator: the teaching scholars program for educators in the health sciences Acad Med 2006 81 969 974 10.1097/01.ACM.0000242593.29279.be 17065858

[B8] Stone S Mazor K Devaney-O‘Neil S Starr S Ferguson W Wellman S Jacobson E Hatem DS Quirk M Development and implementation of an objective structured teaching examination (OSTE) to evaluate improvement in feedback skills following a faculty development workshop Teach Learn Med 2003 15 7 13 10.1207/S15328015TLM1501_03 12632702

[B9] Wipf JE Orlander JD Anderson JJ The effect of a teaching skills course on interns’ and students’ evaluations of their resident-teachers Acad Med 1999 74 938 942 10.1097/00001888-199908000-00021 10495737

[B10] Gruber H Smelser NJ, Baltes PB Acquisition of expertise International encyclopedia of the social and behavioral sciences 2001 Amsterdam: Elsevier 5145 5150

[B11] Lesgold AM Anderson JR, Kosslyn M Acquiring expertise Tutorials in learning and memory 1984 San Francisco: Freeman 31 60

[B12] Lesgold A Rubinson H Feltovich P Glaser R Klopfer D Wang Y Chi MTH, Glaser R, Farr MJ Expertise in a complex skill: diagnosing X-ray pictures The nature of expertise 1988 Hillsdale: Erlbaum 311 342

[B13] Mandl H Gruber H Renkl A Nijhof WJ, Streumer JN Problems of knowledge utilization in the development of expertise Flexibility in training and vocational education 1994 Utrecht: Lemma 291 305

[B14] Boshuizen HPA, Bromme R, Gruber H Gaps and transitions on the way from novice to expert 2004 Dordrecht: Kluwer

[B15] Ericsson KA Deliberate practice and the acquisition and maintenance of expert performance in medicine and related domains Acad Med 2004 79 S70 S81 15383395 10.1097/00001888-200410001-00022

[B16] Julian K Appelle N O‘Sullivan P Morrison EH Wamsley M The impact of an objective structured teaching evaluation on faculty teaching skills Teach Learn Med 2012 24 3 7 10.1080/10401334.2012.641476 22250929

[B17] Beckman TJ Ghosh AK Cook DA Erwin PJ Mandrekar JN How reliable are assessments of clinical teaching? A review of the published instruments J Gen Intern Med 2004 19 971 977 10.1111/j.1525-1497.2004.40066.x 15333063 PMC1492515

[B18] Chitsabesan P Corbett S Walker L Spencer J Barton JR Describing clinical teachers’ characteristics and behaviours using critical incidents and repertory grids Med Educ 2006 40 645 653 10.1111/j.1365-2929.2006.02510.x 16836537

[B19] Copeland HL Hewson MG Developing and testing an instrument to measure the effectiveness of clinical teaching in an academic medical centre Acad Med 2000 75 161 166 10.1097/00001888-200002000-00015 10693849

[B20] Fluit CR Bolhuis S Grol R Laan R Wensing M Assessing the quality of clinical teachers: a systematic review of content and quality of questionnaires for assessing clinical teachers J Gen Intern Med 2010 25 1337 1345 10.1007/s11606-010-1458-y 20703952 PMC2988147

[B21] Hesketh EA Bagnall G Buckley EG A framework for developing excellence as a clinical educator Med Educ 2001 35 555 564 10.1046/j.1365-2923.2001.00920.x 11380858

[B22] Litzelman DK Stratos GA Marriott DJ Skeff KM Factorial evaluation of a widely disseminated educational framework for evaluating clinical teachers Acad Med 1998 73 688 695 10.1097/00001888-199806000-00016 9653408

[B23] Stark R Mandl H Gruber H Renkl A Indeed, sometimes knowledge does not help: a replication study Instr Sci 1998 26 391 407 10.1023/A:1003209514232

[B24] Skeff KM Stratos G Bergen MR Regula DP A pilot study of faculty development for basic science teachers Acad Med 1998 73 701 704 10.1097/00001888-199806000-00018 9653410

[B25] Post RE Quattlebaum RG Benich JJ 3rd Residents-as-teachers curricula: a critical review Acad Med 2009 84 374 380 10.1097/ACM.0b013e3181971ffe 19240450

[B26] Aleamoni LM Student rating myths versus research facts from 1924 to 1998 J Pers Eval Educ 1999 13 2 153 166 10.1023/A:1008168421283

[B27] Fernández J Mateo MA Student and faculty gender in ratings of university teaching quality Sex Roles 1997 37 11–12 997 1003

[B28] McLeod PJ Meagher T Steinert Y Schuwirth L McLeod AH Clinical teachers’ tacit knowledge of basic pedagogic principles Med Teacher 2004 26 23 27 10.1080/01421590310001643154 14744690

[B29] Beckers SK Timmermann A Müller MP Angstwurm M Walcher F Undergraduate medical education in emergency medical care: a nationwide survey at German medical schools BMC Emerg Med 2009 9 7 10.1186/1471-227X-9-7 19435518 PMC2689168

