# Supplementary material for: Does clinical teacher training always improve teaching effectiveness as opposed to no teacher training? A randomized controlled study
Source: BMC Med Educ. 2014 Jan 8;14:6. doi: 10.1186/1472-6920-14-6 (PMC3893403; doi:10.1186/1472-6920-14-6)
Supplement: Additional file 1: Table S1 — Structure of teacher training (overview). [file 1472-6920-14-6-S1.doc]

**Additional file 1: Table S1**: structure of generic teacher training (overview)

| basic didactical training, Charité - University Medicine Berlin | | | | |
| --- | --- | --- | --- | --- |
| **topics** | min | | **contents / formats** | |
| day 1: 8:30 – 16:30 12-14 participants, 1 instructor | | | | |
| **intro** | | 20 | schedule; expectations (moderating chart) | |
| **role of the teacher** | | 40 | reflection on role model ( open discussion) | |
| **needs of learners** | | 60 | types of learners, facilitating understanding | |
| **feedback techniques** | | 60 | feedback (pract. exercise: working in groups) | |
| *lunchtime break (60 min)* | | | | |
| **plenary didactics** | | 150 | short presentations by participants / structure of session / defining learning objectives | |
| **practical skills** | | 60 | teaching of practical skills (Peyton’s 4 steps) | |
| day 2: 8:30 – 16:30 12-14 participants, 2 instructors | | | | |
| **seminar didactics** | | 160 | | excercise: activating learners / structure of session / defining learning objectives (role play / reflection) |
| **theory of learning** | | 20 | | contents as above (summary), interactive lecture |
| *lunchtime break (60 min)* | | | | |
| **teaching with patients** | | 210 | | excercise: bedside teaching / structure of session / defining learning objectives (role play / reflection) |
| **closure / evaluation** | | 30 | | feedback and perspectives |
