# Supplementary material for: Does clinical teacher training always improve teaching effectiveness as opposed to no teacher training? A randomized controlled study
Source: BMC Med Educ. 2014 Jan 8;14:6. doi: 10.1186/1472-6920-14-6 (PMC3893403; doi:10.1186/1472-6920-14-6)
Supplement: Additional file 2: Figure S1 — Questionnaire for evaluation by students. [file 1472-6920-14-6-S2.doc]

**Additional file 2: Figure S1**: Questionnaire for evaluation by students

**Code-nr:**

| **Do you have prior experience in ?**   - Emergency medical service (RettSan, Rett Ass) - Medical assistance professions   - Medical education (Tutor, first-aid-/BLS-instructor) | specify (time in minths) |
| --- | --- |
|  |
| What is your age?  Are you female or male?  Is German your native language?  If not: how long do you already live in Germany? |  |

**very strong/ not at all/**

exellent very poor

|  |  | **+3** | **+2** | **+1** | **0** | **-1** | **-2** | **-3** | **🞸)** |
| --- | --- | --- | --- | --- | --- | --- | --- | --- | --- |
|  | ***How do you rate ...*** |  |  |  |  |  |  |  |  |
|  | ....the general didactical concept ... | ⭘ | ⭘ | ⭘ | ⭘ | ⭘ | ⭘ | ⭘ | ⭘ |
|  | ... quality of teaching structure... | ⭘ | ⭘ | ⭘ | ⭘ | ⭘ | ⭘ | ⭘ | ⭘ |
|  | was the proposed time schedule kept up? | ⭘ | ⭘ | ⭘ | ⭘ | ⭘ | ⭘ | ⭘ | ⭘ |
|  | were learning objectives defined by teachers? | ⭘ | ⭘ | ⭘ | ⭘ | ⭘ | ⭘ | ⭘ | ⭘ |
|  | were the learning objectives helpful to you? | ⭘ | ⭘ | ⭘ | ⭘ | ⭘ | ⭘ | ⭘ | ⭘ |
|  | How much did the teachers follow the learning objectives? | ⭘ | ⭘ | ⭘ | ⭘ | ⭘ | ⭘ | ⭘ | ⭘ |
|  | How much was my personal learning situation respected? | ⭘ | ⭘ | ⭘ | ⭘ | ⭘ | ⭘ | ⭘ | ⭘ |
|  | To what extent did I acquire practical skills? | ⭘ | ⭘ | ⭘ | ⭘ | ⭘ | ⭘ | ⭘ | ⭘ |
|  | To what extent did I change attitudes? | ⭘ | ⭘ | ⭘ | ⭘ | ⭘ | ⭘ | ⭘ | ⭘ |
|  | How well do I feel prepared for an emergency in real life? | ⭘ | ⭘ | ⭘ | ⭘ | ⭘ | ⭘ | ⭘ | ⭘ |

**very strong/ not at all/**

**exellent very poor**

|  | **The teachers .....** | **+3** | **+2** | | **+1** | **0** | | **-1** | **-2** | | **-3** | ***)** | |
| --- | --- | --- | --- | --- | --- | --- | --- | --- | --- | --- | --- | --- | --- |
|  | ....were able to pass on learning contents | ⭘ | ⭘ | | ⭘ | ⭘ | | ⭘ | ⭘ | | ⭘ | ⭘ | |
| 1. o | ....could be understood clearly | ⭘ | ⭘ | | ⭘ | ⭘ | | ⭘ | ⭘ | | ⭘ | ⭘ | |
|  | ....were able to highlight important information | ⭘ | ⭘ | | ⭘ | ⭘ | | ⭘ | ⭘ | | ⭘ | ⭘ | |
|  | ....showed enthusiasm | ⭘ | ⭘ | | ⭘ | ⭘ | | ⭘ | ⭘ | | ⭘ | ⭘ | |
|  | ... answered questions in competent manner | ⭘ | ⭘ | | ⭘ | ⭘ | | ⭘ | ⭘ | | ⭘ | ⭘ | |
|  | ....supported contributions made by students | ⭘ | ⭘ | | ⭘ | ⭘ | | ⭘ | ⭘ | | ⭘ | ⭘ | |
|  | ....provided valuable feedback concerning my practical skills | ⭘ | ⭘ | | ⭘ | ⭘ | | ⭘ | ⭘ | | ⭘ | ⭘ | |
|  | ....chose an adequate pace of presentation | ⭘ | ⭘ | | ⭘ | ⭘ | | ⭘ | ⭘ | | ⭘ | ⭘ | |
|  | ....made appropriate use of media | ⭘ | ⭘ | | ⭘ | ⭘ | | ⭘ | ⭘ | | ⭘ | ⭘ | |
|  | ....started sessions in time | ⭘ | ⭘ | | ⭘ | ⭘ | | ⭘ | ⭘ | | ⭘ | ⭘ | |
|  | ... finished sessions in time | ⭘ | ⭘ | | ⭘ | ⭘ | | ⭘ | ⭘ | | ⭘ | ⭘ | |
|  | ... summarised points of importance | ⭘ | ⭘ | | ⭘ | ⭘ | | ⭘ | ⭘ | | ⭘ | ⭘ | |
|  | ....provided problem solving skills | ⭘ | ⭘ | | ⭘ | ⭘ | | ⭘ | ⭘ | | ⭘ | ⭘ | |
|  | ....established a contact between teachers and learners? | ⭘ | ⭘ | | ⭘ | ⭘ | | ⭘ | ⭘ | | ⭘ | ⭘ | |
| **Global acceptance of teachers** | | | | | | | | | | | | | |
|  | Course director (Senior) | ⭘ | ⭘ | | ⭘ | ⭘ | | ⭘ | ⭘ | | ⭘ | ⭘ | |
|  | 2nd anesthesist (intermediate) | ⭘ | ⭘ | | ⭘ | ⭘ | | ⭘ | ⭘ | | ⭘ | ⭘ | |
|  | Junior anesthesist | ⭘ | ⭘ | | ⭘ | ⭘ | | ⭘ | ⭘ | | ⭘ | ⭘ | |
|  | my overall acceptance of the course was … | ⭘ | ⭘ | | ⭘ | ⭘ | | ⭘ | ⭘ | | ⭘ | ⭘ | |
|  | How well did I feel prepared for a real life emergency before this course started? | ⭘ | | ⭘ | ⭘ | | ⭘ | ⭘ | | ⭘ | ⭘ | | ⭘ |
|  | I was able to identify with the teachers | ⭘ | | ⭘ | ⭘ | | ⭘ | ⭘ | | ⭘ | ⭘ | | ⭘ |

*Svensson, Siggemann, Breckwoldt*
