# Supplementary material for: Does clinical teacher training always improve teaching effectiveness as opposed to no teacher training? A randomized controlled study
Source: BMC Med Educ. 2014 Jan 8;14:6. doi: 10.1186/1472-6920-14-6 (PMC3893403; doi:10.1186/1472-6920-14-6)
Supplement: Additional file 3: Table S2 — Characteristics of students. [file 1472-6920-14-6-S3.doc]

**Additional file 3: Table S2**: characteristics of students

| n = 193 | total | teachers  **with**  **training**  n = 96 | teachers  **without**  **training**  n = 97 | statistical  difference |
| --- | --- | --- | --- | --- |
| age | 23 (22-26) | 24 (23-27) | 23 (22-26) | n.s. |
| gender female | 58.5% | 56.3% | 59.6% | n.s. |
| previous experience in EMS ^1)^ | 24 (12.4%) | 13 (13.5%) | 11 (11.3%) | n.s. |
| native language German | 86.0% | 85.4% | 86.6% | n.s. |

^1)^ emergency medical service
