# Supplementary material for: Does clinical teacher training always improve teaching effectiveness as opposed to no teacher training? A randomized controlled study
Source: BMC Med Educ. 2014 Jan 8;14:6. doi: 10.1186/1472-6920-14-6 (PMC3893403; doi:10.1186/1472-6920-14-6)
Supplement: Additional file 4: Figure S2 — Resulting final study configuration. [file 1472-6920-14-6-S4.doc]

**Additional file 4: Figure S2**: resulting final study setup / flow chart / constellation
