# Supplementary material for: Does clinical teacher training always improve teaching effectiveness as opposed to no teacher training? A randomized controlled study
Source: BMC Med Educ. 2014 Jan 8;14:6. doi: 10.1186/1472-6920-14-6 (PMC3893403; doi:10.1186/1472-6920-14-6)
Supplement: Additional file 5: Table S3 — MCQ by single questions. [file 1472-6920-14-6-S5.doc]

**Additional file 5: Table S3**: detailed results of MCQ

| **MCQ, detailed results of specific questions** | | | | | |
| --- | --- | --- | --- | --- | --- |
|  | **objective of question** | **type of question** ^1)^ | **results**  (% of correct answers) | | **p-value** |
|  |  |  | **trained**  **teachers** | **untrained**  **teachers** |  |
| 1 | chest compressions | **c** | **57.3** | **77.3 ^*)^** | **0.020** |
| 2 | cardiac arrest medication | **c** | **21.9 ^*)^** | **08.2** | **0.047** |
| 3 | chest compressions | **c** | 55.2 | 60.8 | n.s. |
| 4 | chest compressions | **c** | 89.6 | 83.0 | n.s. |
| 5 | cardiac arrest rhythms | **c** | 75.0 | 82.0 | n.s. |
| 6 | ECG monitoring | **p** | 86.5 | 86.0 | n.s. |
| 7 | AED principles | **c** | **72.9** | **90.0** ^*)^ | **0.032** |
| 8 | recovery position | **c** | 94.8 | 95.0 | n.s. |
| 9 | trauma / unconscious | **p** | 81.3 | 89.0 | n.s. |
| 10 | airway mangement | **c** | **33.3** | **45.0** ^*)^ | **0.010** |
| 11 | defibrillation | **c** | **82.3** | **90.0** ^*)^ | **0.015** |
| 12 | unconsciousness | **p** | 67.7 | 57.0 | n.s. |
| 13 | activating help | **c** | 87.5 | 78.0 | n.s. |
| 14 | unconsciousness / BLS | **p** | **74.0** | **84.0** ^*)^ | **0.013** |
|  | **overall score** |  | **71.4%** | **78.6%** | **0.075** |

^1)^ type of question (c: cognitive, p: procedural)

^*)^ significantly higher value than compared group (< 0.05)
